# Comparative Analysis of Soil Microbial Community Structures in Rhizosphere of Two Texture-Differentiated Lotus Root Varieties

**DOI:** 10.3390/microorganisms13071637

**Published:** 2025-07-10

**Authors:** Xinni Li, Qiyue Liang, Meiping Gao, Yangxiu Ou, Yifeng Hu, Wen Jiang, Huiping Jiang, Shangdong Yang

**Affiliations:** 1 Guangxi Key Laboratory of Agro-Environment and Agro-Products Safety, National Demonstration Center for Experimental Plant Science Education Guangxi Agricultural College, Guangxi University, Nanning 530004, China; lixinni19@163.com (X.L.); 19994695054@163.com (Q.L.); 2 Vegetable Research Institute, Guangxi Academy of Agricultural Sciences, Nanning 530007, China; gmp2009@163.com (M.G.); oyx1322@139.com (Y.O.); sunmoonfeng@126.com (Y.H.); jiangwen5476581@163.com (W.J.)

**Keywords:** lotus root, rhizosphere, microbial compositions, polysaccharides, texture variation

## Abstract

To investigate the relationship between the rhizosphere microbial community structure and lotus root texture, the biological properties, and the rhizosphere microbial composition of mealy (ML) and crunchy lotus (CL) varieties were all analyzed using traditional and high-throughput sequencing technologies. The results showed that the ML varieties exhibited significantly lower moisture but higher starch contents than those of CL. Meanwhile, the rhizosphere fungal richness of ML was also significantly higher than that of CL. Moreover, the relative abundances of bacterial phyla and genera, such as Nitrospirota, Bacteroidota, Proteobacteria, and *Bacillus*, alongside fungal phyla and genera, i.e., Ascomycota and *Emericellopsis*, were enriched in rhizosphere of ML compared to CL. Functional prediction also revealed that elevated nitrogen cycling, polysaccharide degradation and cellulose breakdown functions could be detected in ML, potentially driving starch accumulation and cell wall modification. These results suggest that rhizosphere microbial composition, particularly nitrogen-cycling bacteria and lignocellulose-degrading fungi, may contribute to texture formation between texture-differentiated lotus root varieties.

## 1. Introduction

Lotus root (*Nelumbo nuciferais* Gaertn.), a perennial aquatic herb belonging to the family Nymphaeaceae [1], is widely cultivated as a nutrient-rich vegetable in China, Australia, India, and Japan [2]. Its rhizomes contain diverse bioactive compounds, including carbohydrates (starch, dietary fiber, and sugars), proteins, amino acids, minerals, vitamins, and phenolic compounds [3,4,5,6]. These components contribute to various health benefits, such as antidiarrheal [7], antimicrobial [8], hypotensive [9], hypocholesterolemic [10], hypoglycemic [11], hepatic steatosis-alleviating [12], and diuretic effects [13]. With increasing public awareness of its nutritional value, lotus root has gained significant agricultural importance. However, the textural characteristics (e.g., mealy vs. crunchy) of different varieties, which critically influence consumer acceptance and processing, remain insufficiently understood. Investigating the factors driving texture variation—particularly the potential role of rhizosphere microbial communities—is essential for optimizing breeding programs, and enhancing market competitiveness.

Texture is one of the key characteristics of food and is often used as an important indicator for assessing the quality and pricing of vegetables and fruits [14]. This texture is closely related to the structural integrity of plant cell walls, which are primarily composed of cellulose, hemicellulose, lignin, and pectin, along with smaller amount of proteins (such as structural glycoproteins), lipids, and other substances [15]. Among them, cellulose, as a stable polysaccharide, forms the primary structural framework of the cell wall, providing rigidity and resistance, while hemicellulose enhances flexibility by cross-linking cellulose microfibrils. Also, the integrity of the cell wall, maintained by these components, critically influences the mechanical properties of lotus root by resisting cellular rupture during processing [16]. Additionally, the texture is modulated by internal components, such as starch content, starch granule morphology, and moisture content [17]. Based on texture, lotus root can be classified into two types. The first type is mealy lotus root, characterized by a high starch content (predominantly amylopectin) and low moisture content. Upon cooking, the starch fully gelatinizes, forming a soft, sticky, and cohesive texture. This type exhibits high thermal stability, making it ideal for boiling, or steaming. The second type is crunchy lotus root, which contains lower starch but high moisture and soluble sugars. During processing, limiting starch gelatinization occurs due to competitive water binding by sugars, followed by rapid retrogradation. This results in minimal viscosity and a crisp, refreshing texture, suited for cold dishes or quick stir-frying [17,18].

The composition and function of the rhizosphere soil microbial communities are strongly influenced by plant variety [19,20]. Through species-specific root exudates (e.g., organic acids and secondary metabolites), different plant varieties selectively shape microbial assembles in the rhizosphere, leading to distinct community structures and metabolic potentials [21,22,23]. For instance, disease-resistant strawberry cultivars enrich beneficial microbes (e.g., *Pseudomonas* spp.) that suppress soil-borne pathogens, highlighting the link between microbial recruitment and plant fitness [24]. These microbiota, in turn, interact with plants by producing phytoactive compounds [25]. Certain rhizobacteria synthesize auxins [26] and cytokinins [27], which directly promote root growth, while others modulate plant stress responses through ethylene [28] or abscisic acid [29] signaling. Additionally, microbial synthesis of jasmonic acid analogs or induction of salicylic acid pathways in plants further fine-tunes immune responses [30]. Collectively, these interactions optimize the rhizosphere environment, enhancing nutrient acquisition and stress resilience [31].

Current research on lotus root primarily focuses on cultivation and breeding [32,33], nutritional profiling [34], and physiological functions [35]. However, studies investigating microbial contributions to lotus root quality formation remain scarce. To address this gap, we propose that the textural differences between mealy (ML) and crunchy (CL) lotus root varieties are closely associated with the microbial composition and function in rhizosphere. To test this hypothesis, soil microbial community structures in the rhizosphere of ML and CL root varieties are analyzed. Our goal specifically is to examine how microbial community structures potentially regulate textural differentiation.

## 2. Materials and Methods

### 2.1. Field Site Description and Experimental Designs

The experiment was conducted at the Li Jian Scientific Experimental Site of the Guangxi Academy of Agricultural Sciences (108°17′ E, 23°25′ N). The soil at the experimental site was classified as red loam with pH 5.49. The total nitrogen, phosphorus, and potassium contents were 0.86, 0.46, and 2.73 g·kg^−1^, respectively. Additionally, the available nitrogen, phosphorus, and potassium contents were 54.5, 8.7, and 91.0 mg·kg^−1^, respectively.

A randomized complete block design with three biological replicates was implemented. And six lotus root varieties were selected based on textural phenotypes: mealy lotus root varieties (Qintang, Red beauty, Baiyuzan; abbreviated, ML group) and crunchy lotus root varieties (Elian 6, Elian 10, Dabaipang; abbreviated, CL group). All varieties were simultaneously sown and grown in the same field on 10 April 2023, i.e., each variety was randomly planted in a 500 m^2^ pond (length is 100 m and width is 50 m). All varieties were managed under identical conditions.

### 2.2. Soil Samples Collection

Samples were randomly collected in November 2023. The soil surrounding each plant was loosened using a spade disinfected with 75% ethanol spray, and the entire plant was carefully pulled out by firmly grasping its base and manually extracting it. The roots were then rinsed with sterile water to remove surface debris. Meanwhile, soil samples from the same field, managed under identical conditions but without lotus root plants, were collected as background samples (CK). These samples were placed in sterile plastic bags, packed with ice in a polystyrene foam box and transported to the laboratory as soon as possible and sieved using a 2 mm stainless steel mesh and stored at −80 °C.

### 2.3. Test Methods

The moisture content was measured using a Nexus FT-IR spectrometer (Thermo Nicolet Corporation, Waltham, MA, USA) within the spectral range of 4000–10,000 cm [36]. Starch was separated using absolute ethanol [37].

Total DNA extraction, PCR amplification, and sequence determination of the soil samples were performed by Shanghai Majorbio Biopharm Technology Co., Ltd., Shanghai, China. High-throughput sequencing was performed using the MiSeq platform.

Total DNA extraction was performed based on the instructions of the FastDNA^®^ Spin Kit for Rhizosphere (MP Biomedicals, Santa Ana, CA, USA), while DNA concentration and purity were measured using a NanoDrop 2000 spectrophotometer (Thermo Fisher Scientific, Waltham, MA, USA). The ABI GeneAmp^®^ type 9700 (ABI, Carlsbad, CA, USA) was used for the PCR, and the products were recovered by 2% agarose gel electrophoresis and purified by an AxyPrep. Their specific primers and sequence types are shown in Table 1.

Illumina MiSeq sequencing: PCR products from the same sample were purified using the AxyPrep DNA Gel Extraction Kit (Axygen Biosciences, Union City, CA, USA), mixed and detected by recovery using a 2% agarose gel. The recovered products were quantified using a Quantus™ Fluorometer (Promega, Madison, WI, USA). Library construction was performed using the NEXTFLEX^®^ Rapid DNA-Seq Kit.

Sequencing was performed using the Illumina Miseq platform (Shanghai Majorbio Bio-pharm Technology Co., Ltd., Shanghai, China). The raw data were uploaded to the NCBI database for comparison.

### 2.4. Statistical Analyses

The experimental data underwent statistical analysis using Excel 2019 and SPSS Statistics 27 software. Data are presented as the mean ± standard deviation (SD). The significance analyses were conducted using the Wilcoxon rank sum test, with microbial diversity and richness expressed as Shannon and Simpson, Ace, and Chao1 indices, respectively. Online data analysis was performed using the I-sanger cloud data analysis platform provided by Shanghai Majorbio Bio-Pharm Technology Co., Ltd., Shanghai, China.

## 3. Results

Raw data from sequencing of rhizospheres bacteria and fungi of lotus root have been deposited in the NCBI database under accession numbers PRJNA1275841 and PRJNA1275856, respectively.

### 3.1. Biological Characteristics of Different Varieties of Lotus Roots

**Figure 1:**
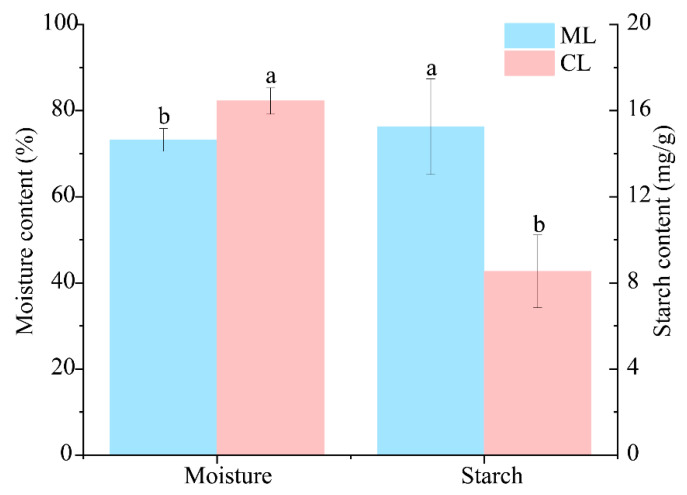
Biological characteristics of different varieties of lotus roots. ML: mealy lotus varieties; CL: crunchy lotus varieties. All data are presented as the mean ± SD (standard deviation). Different letters in the column indicate significant differences among treatments at *p* < 0.05.

As seen in Figure 1, the moisture content in the meany lotus root varieties (ML) was significantly lower than that in the crunchy lotus root varieties (CL). However, the starch content was significantly higher than that in the crunchy lotus root varieties (CL).

### 3.2. Soil Microbial Diversity in Rhizospheres in Mealy and Crunchy Lotus Root Varieties

As shown in Table 2, the coverage indices reached over 96%, indicating that the sequencing data were reliable. Comparative analysis also revealed that there were no significant differences in soil bacterial diversity (Shannon and Simpson indices) and richness (Ace and Chao1 indices) between ML and CL. Additionally, no significant differences in rhizosphere between ML and CL regarding soil fungal diversity, whereas soil fungal richness in rhizosphere of ML was significantly higher than that in CL.

**Figure 2:**
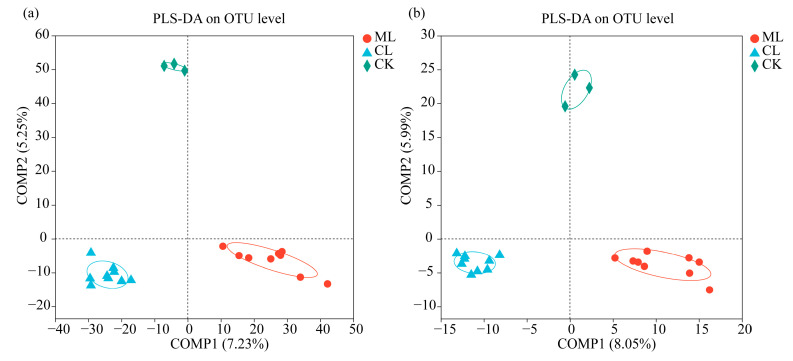
Comparison of the soil bacterial and fungal compositions among ML, CL, and CK. PLS-DA score plot of the soil bacterial (**a**) and fungal (**b**) communities among ML, CL, and CK. ML: mealy lotus varieties; CL: crunchy lotus varieties.

At the OTU level, the results of Partial Least Squares Discriminant Analysis (PLS-DA) revealed that distinct clustering of soil bacterial (Figure 2a) and fungal (Figure 2b) communities could all be detected among ML, CL, and CK. These results indicate that soil bacterial and fungal compositions in rhizosphere show significant differences among ML, CL, and CK. In other words, the mealy and crunchy lotus root varieties recruited different soil bacterial and fungal communities in their rhizosphere.

### 3.3. Soil Microbial Compositions of Rhizospheres in Mealy and Crunchy Lotus Root Varieties

**Figure 3:**
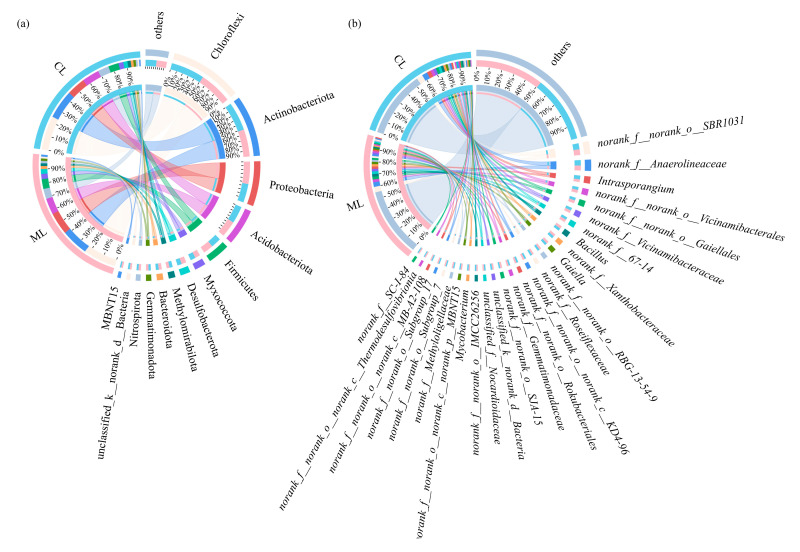

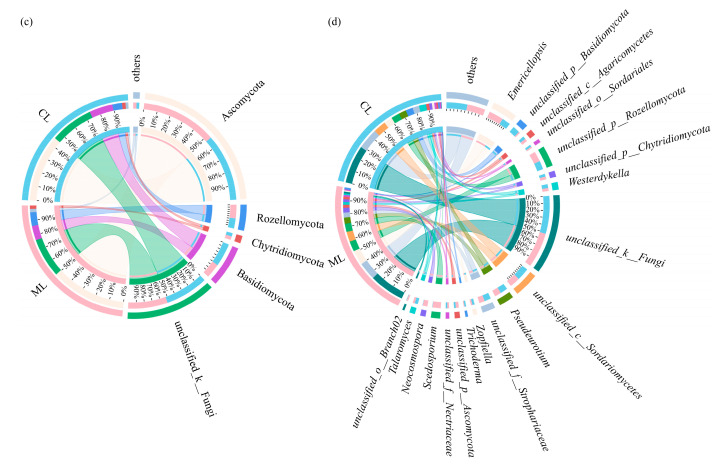
(**a**) Compositions of rhizosphere soil bacterial communities at the phylum level; (**b**) compositions of rhizosphere soil bacterial communities at the genus level. (**c**) Compositions of rhizosphere soil fungal communities at the phylum level; (**d**) compositions of rhizosphere soil fungal communities at the genus level. ML: mealy lotus varieties, CL: crunchy lotus varieties.

As shown in Figure 3a, Chloroflexi, Actinobacteriota, Proteobacteria, Acidobacteriota, Firmicutes, Myxococcota, Methylomirabilota, Bacteroidota, Gemmatimonadota, Nitrospirota, and unclassified_k__norank_d__Bacteria were the dominant soil bacterial phyla (i.e., relative abundance > 1%) in rhizosphere for both ML and CL. Among the bacterial phyla grouped as “others”, Planctomycetota was the most abundant in the rhizosphere of both ML and CL, although its relative abundance was below the 1% threshold. Moreover, the abundances of Nitrospirota were significantly higher in rhizospheres of ML than those of CL. Furthermore, it is noteworthy that MBNT15 was a unique dominant soil bacterial phylum in ML. 

At the genus level, 22 and 24 dominant bacterial genera could be detected in rhizospheres of ML and CL, respectively. Among them, *norank_f__norank_o__SBR1031*, *norank_f__Anaerolineaceae*, *Intrasporangium*, *norank_f__norank_o__Vicinamibacterales*, *norank_f__Vicinamibacteraceae*, *norank_f__norank_o__Gaiellales*, *norank_f__67-14*, *Bacillus*, *norank_f__Xanthobacteraceae*, *Gaiella*, *norank_f__norank_o__RBG-13-54-9*, *norank_f__norank_o__Rokubacteriales*, *norank_f__norank_o__norank_c__KD4-9*, *norank_f__Roseiflexaceae*, *norank_f__Gemmatimonadaceae*, *unclassified_k__norank_d__Bacteria*, *norank_f__norank_o__SJA-15*, *norank_f__norank_o__IMCC26256,* and *Mycobacterium* were the common dominant bacterial genera in rhizospheres of ML and CL (Figure 3b). Additionally, within the “others” bacterial genera grouped, *norank_f__A4b* showed the highest relative abundance in both ML and CL rhizospheres. Moreover, *norank_f__norank_o__norank_c__norank_p__MBNT15*, *norank_f__norank_o__norank_c__Thermodesulfovibrionia*, and *norank_f__SC-I-84* were the unique dominant soil bacterial genera in the rhizosphere of ML. By contrast, *unclassified_f__Nocardioidaceae*, *norank_f__norank_o__norank_c__MB-A2-108*, *norank_f__Methyloligellaceae*, *norank_f__norank_o__Subgroup_7*, and *norank_f__norank_o__Subgroup_17* were the unique dominant soil bacterial genera in the rhizosphere of CL.

Additionally, a total of five dominant fungal phyla, i.e., Ascomycota, unclassified_k__Fungi, Basidiomycota, Rozellomycota, and Chytridiomycota could be detected in rhizospheres of ML and CL (Figure 3c). Among the “others” fungal phyla, Glomeromycota was the most abundant in both ML and CL rhizospheres.

At the genus level, 15 and 16 dominant fungal genera could be detected in rhizospheres of ML and CL, respectively. Among them, *unclassified_k__Fungi*, *unclassified_c__Sordariomycetes*, *Emericellopsis*, *unclassified_p__Rozellomycota*, *Pseudeurotium*, *unclassified_f__Strophariaceae*, *Westerdykella*, *Talaromyces*, *unclassified_c__Agaricomycetes*, *unclassified_p__Chytridiomycota*, *unclassified_p__Ascomycota*, and *unclassified_f__Nectriaceae* were the common dominant fungal genera in the rhizospheres of MS and CL (Figure 3d). In addition, among the “others” fungal genera grouped, *Zopfiella* showed the highest relative abundance in ML, while *unclassified_o__Agaricales* was the most abundant in CL. Moreover, *Scedosporium*, *Neocosmospora*, and *unclassified_o__Branch02* were the unique dominant soil fungal genera in rhizosphere of ML, whereas *unclassified_p__Basidiomycota*, *Zopfiella*, *unclassified_o__Sordariales*, and *Trichoderma* were the unique dominant soil fungal genera in the rhizosphere of CL.

Linear Discriminant Analysis Effect Size (LEfSe) analysis with a linear discriminant analysis (LDA) threshold of 1.0 was also used to identify significant differences and primary influential biomarker categories in the rhizosphere between ML and CL.

**Figure 4:**
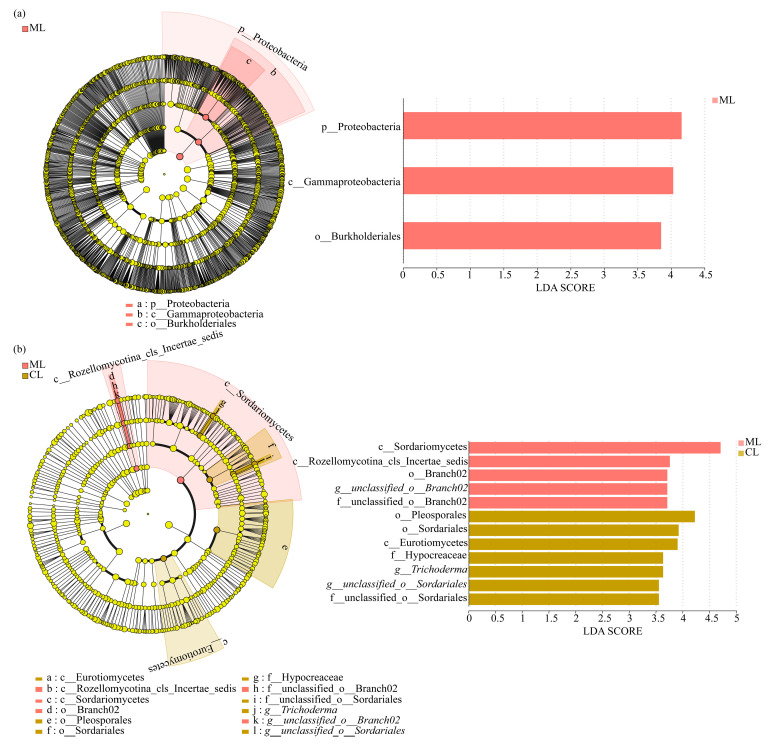
LEfSe analysis of soil bacteria (**a**) and fungi (**b**) in rhizospheres between ML and CL varieties. ML: mealy lotus varieties; CL: crunchy lotus varieties.

As shown in Figure 4a, at the phylum level, Proteobacteria was significantly clustered in the rhizospheres of ML.

Moreover, unclassified_o__Branch02 was significantly enriched in the rhizospheres of ML, whereas Trichoderma and unclassified_o__Sordariales were significantly enriched in the rhizospheres of CL (Figure 4b).

### 3.4. Venn Diagram Analysis of Soil Microorganisms in Rhizosphere of Mealy and Crunchy Lotus Root Varieties

**Figure 5:**
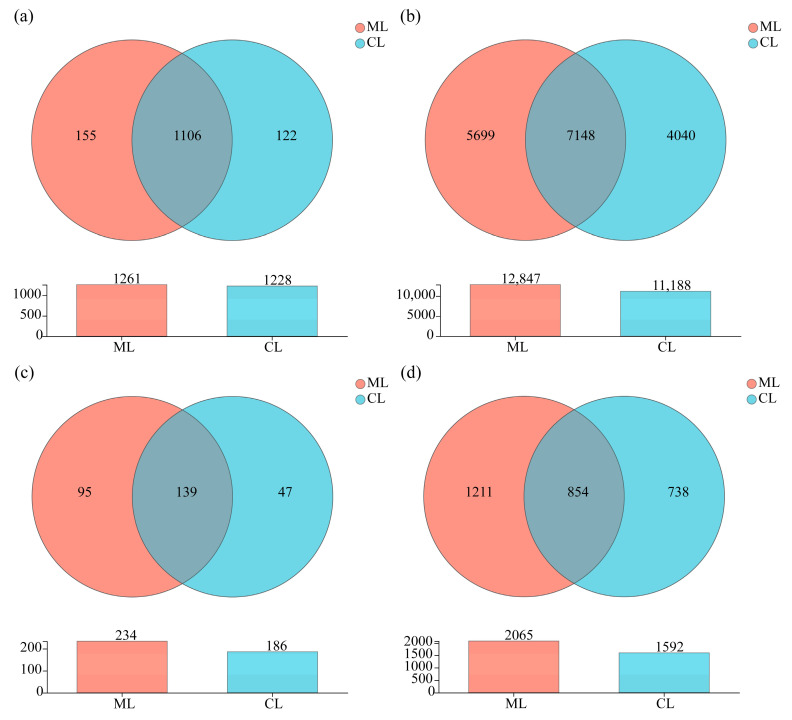
(**a**) Venn diagram analyses of soil bacteria at the genus level. (**b**) Venn diagram analyses of soil bacteria at the OTU level. (**c**) Venn diagram analyses of soil fungi at the genus level. (**d**) Venn diagram analyses of soil fungi at the OTU level. ML: mealy lotus varieties; CL: crunchy lotus varieties.

At the genus level, the numbers of unique soil bacterial genera in the rhizosphere of ML and CL varieties were 155 and 122, respectively, and the total number of corresponding soil bacterial genera was 1261 and 1228 (Figure 5a). Moreover, 12,847 and 11,188 bacterial OTUs were found in ML and CL soil, respectively. Among them, 7148 common soil-bacterial OTUs were detected in both rhizospheres, and 5699 and 4040 unique soil bacterial OTUs were detected in the rhizospheres of ML and CL, respectively (Figure 5b).

Furthermore, the numbers of unique soil fungal genera in the rhizosphere of ML and CL were 95 and 47, respectively, and the total numbers of soil fungi genera in the rhizosphere of ML and CL were 234 and 186, respectively (Figure 5c). A total of 854 common soil fungal OTUs were identified, while 1211 and 738 unique OTUs were found in the rhizosphere of ML and CL, respectively (Figure 5d).

All of the above results suggested that different textural lotus varieties (ML and CL) recruited different soil microorganisms in the rhizosphere. In comparison with CL, higher abundance of bacteria and fungi needed to be recruited in the rhizosphere of ML.

### 3.5. Functional Predictions of Soil Microbial Communities in Rhizospheres of Mealy and Crunchy Lotus Root Varieties

**Figure 6:**
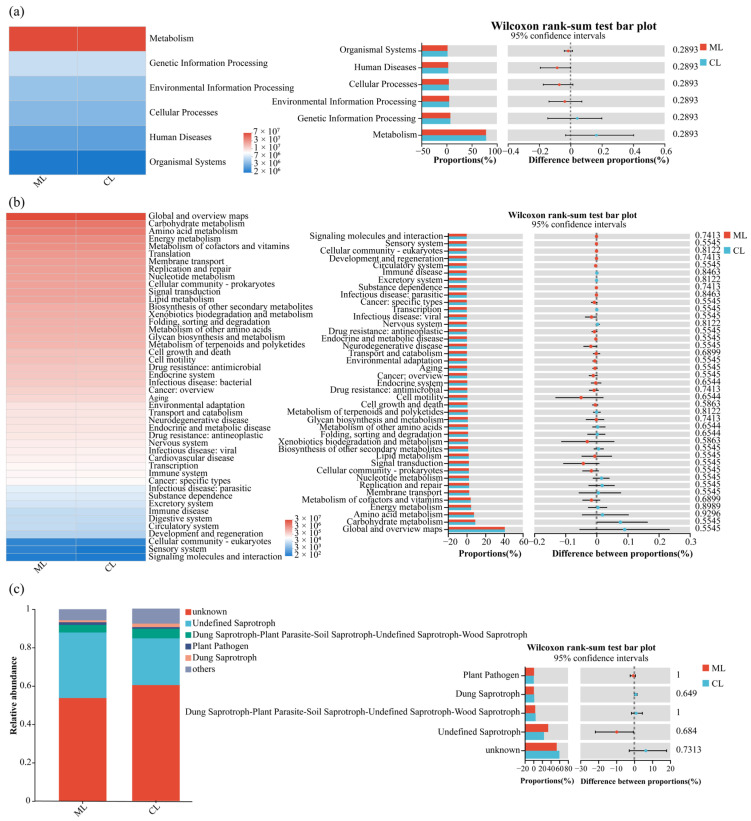
Functional predictions of soil bacterial and fungal communities in rhizospheres of ML and CL. Compositional variability test for bacterial (**a**,**b**) and fungal (**c**) communities. ML: mealy lotus varieties; CL: crunchy lotus varieties.

Based on the KEGG (Kyoto Encyclopedia of Genes and Genomes) database, six metabolic pathways of soil bacterial communities were identified in the rhizospheres of ML and CL, i.e., they were metabolism, genetic information processing, environmental information processing, cellular processes, human diseases, and organismal systems (Figure 6a). However, the Wilcoxon rank-sum test also indicated that there were no significant differences in the relative abundances of these pathways between ML and CL.

Moreover, a total of 46 bacterial metabolic pathways were detected in the rhizospheres of ML and CL. These pathways encompassed global and overview maps, carbohydrate metabolism, amino acid metabolism, energy metabolism, and the metabolism of cofactors and vitamins, among others (Figure 6b). Comparative analysis revealed that the overall functional composition of bacterial communities was largely conserved between the two varieties with no significant enrichment or depletion of specific pathways.

Based on FUNGuild functional predictions, five major fungal ecological categories were identified in the rhizosphere, including undefined saprotrophs, dung saprotroph-plant, parasite-soil saprotroph-undefined saprotroph-wood saprotroph, plant pathogens, and dung saprotrophs (Figure 6c). The Wilcoxon rank-sum test indicated that there were no significant differences in the five ecological functional categories fungal communities in the rhizosphere between ML and CL varieties.

## 4. Discussions

The textural quality of lotus root, a crucial aquatic vegetable, is predominantly determined by its cellular composition. The cell walls of higher plants are composed of polysaccharides such as pectin, cellulose, and hemicellulose [38]. Kan Juan et al. [39] demonstrated that the enzymatic degradation of pectic backbone and neutral sugars side-chains (e.g., galactose) in cell wall polysaccharides critically contributes to tissue softening through the action of polysaccharide-degrading enzymes. This process is further regulated by differential expression of cell wall-modifying enzyme genes during ripening [40]. Complementing these structural factors, intracellular components markedly influence textural properties. Starch content shows a strong negative correlation with crispness, where mealy lotus roots contain higher starch content compared to lower starch content in crunchy lotus roots [41].

As root exudates and nutrient composition can shape soil microbial communities in the rhizosphere of plants [42]. Our results revealed that not only soil bacterial and fungal composition in the rhizosphere of mealy (ML) were significantly different compared to crunchy lotus (CL) varieties, but also found that soil fungal richness in the rhizosphere of ML was significantly higher than that of CL, i.e., it also indicated that different genotypes of lotus root selectively recruited distinct bacterial and fungal communities in the rhizosphere. In addition, the rhizosphere microbial communities of both ML and CL differed markedly from those in the background soil (CK), highlighting the influence of plant genotype on microbial recruitment. PLS-DA analysis confirmed clear separation among ML, CL, and CK, suggesting that lotus root varieties could significantly shape their rhizosphere microbiota.

In comparison with CL varieties, the relative abundance of Nitrospirota was significantly higher in the rhizosphere of ML (1.74%) than in that of CL (1.24%). Similarly, Bacteroidota also showed a higher abundance in ML (2.15%) compared to CL (1.89%). Meanwhile, LEfSe analysis also revealed that Proteobacteria significantly enriched in the ML rhizosphere. As Nitrospirota participates in nitrogen cycle by oxidizing nitrite to nitrate, with nitrate being one of the nitrogen sources available to plants [43]. Moreover, some strains of Proteobacteria are known nitrifying bacteria, capable of oxidizing ammonia to nitrite and further oxidizing nitrite to nitrate [44]. And adequate nitrogen supply can increase photosynthetic rate and starch accumulation [45]. Bacteroidota is a major degrader of polysaccharides, and its genome contains a large number of carbohydrate-active enzymes (CAZymes), which are arranged in polysaccharide utilization loci (PULs) for degrading plant polysaccharides [46]. Furthermore, Bacillus was the dominant soil bacterial genus in the rhizosphere of ML compared to CL varieties. As Bacillus can produce cellulases with high activity and stability under different environmental conditions, it also suggests that Bacillus may contribute to cellulose degradation [47].

Although Ascomycota, unclassified_k__Fungi, Basidiomycota, Rozellomycota, and Chytridiomycota were all the dominant fungal phyla in the rhizosphere of ML and CL. However, their relative abundances were quite different in ML and CL. For instance, the abundance of Ascomycota was higher in ML (50.47%) than in CL (45.08%). Ascomycota sp. SM2 had been demonstrated to process extremely high carboxymethyl cellulase and filter paper cellulase activities [48]. At genus level, *Emericellopsis* was also more abundant in the rhizosphere of ML (11.07%) compared to CL (6.39%). *Emericellopsis cladophorae* can secrete a variety of carbohydrate-active enzymes, such as cellulases, chitinases, and pectinases, which play an important role in degrading polysaccharides, such as starch and cellulose [49].

Additionally, based on the KEGG database, similar metabolic pathways of soil microbial communities, such as carbohydrate metabolism, amino acid metabolism, and energy metabolism were detected in the rhizosphere of ML and CL. FUNGuild functional prediction also found that no significant differences in soil fungal ecological functional categories in rhizosphere between ML and CL. However, the enrichment of special dominant soil microorganisms in the rhizosphere of ML variety also indicated that its potential advantage of soil microorganisms in starch metabolism and cellulose degradation. Notably, rhizosphere microbes in ML enhanced polysaccharide degradation and cellulose breakdown likely weaken cell-wall rigidity (e.g., pectin and hemicellulose networks), facilitating starch granule expansion and a softer, cohesive “mealy” texture.

## 5. Conclusions

Mealy lotus (ML) varieties exhibited significantly lower moisture but higher starch contents than those of crunchy lotus (CL) varieties. Meanwhile, rhizosphere fungal richness of ML was also significantly higher than that of CL. Moreover, the relative abundances of bacterial phyla and genera, such as Nitrospirota, Bacteroidota, Proteobacteria, and *Bacillus*, alongside fungal phyla and genera, i.e., Ascomycota and *Emericellopsis* were enriched in the rhizosphere of ML compared to CL. Functional prediction also revealed that elevated nitrogen cycling, polysaccharide degradation and cellulose breakdown functions could be detected in ML, potentially driving starch accumulation and cell wall modification. These results suggest that rhizosphere microbial composition, particularly nitrogen-cycling bacteria and lignocellulose-degrading fungi may contribute to texture formation between texture-differentiated lotus root varieties.

## Figures and Tables

**Table 1:** Sequence types and primer sequences.

Sequence Type	Primer Name	Primer Sequence	Length Sequencing	Sequencing Platform
Bacterial 16S rRNA	338F	5′-ACTCCTACGGGAGGCAGCAG-3′	416 bp	Miseq PE300
	806R	5′-GGACTACHVGGGTWTCTAAT-3′		
Fungal ITS	ITS1F	5′-CTTGGTCATTTAGAGGAAGTAA-3′	251 bp	MiSeq PE300
	ITS2R	5′-GCTGCGTTCTTCATCGATGC-3′		

**Table 2:** Soil microbial diversity in rhizospheres of mealy and crunchy lotus root varieties.

	Samples	Shannon Index	Simpson Index	Ace Index	Chao 1 Index	Coverage
Bacteria	ML	7.17 ± 0.07a	0.0024 ± 0.0003a	5847.07 ± 325.66a	5506.40 ± 290.86a	0.96
	CL	7.04 ± 0.26a	0.0041 ± 0.0051a	5587.18 ± 286.22a	5294.94 ± 247.26a	0.96
	CK	7.13 ± 0.01a	0.0024 ± 0.0002a	5895.46 ± 81.78a	5551.98 ± 47.85a	0.96
Fungi	ML	4.33 ± 0.71a	0.069 ± 0.0948a	556.56 ± 114.02a	559.30 ± 113.97a	0.99
	CL	4.47 ± 0.31a	0.0356 ± 0.0186a	385.24 ± 89.06b	387.66 ± 89.26b	0.99
	CK	4.43 ± 0.35a	0.0471 ± 0.0386a	417.13 ± 80.85ab	419.51 ± 80.97ab	0.99

Notes: Data in the table are presented as the means ± SDs; values followed by different lowercase letters indicate significant differences at *p* < 0.05 among ML, CL, and CK (ML: mealy lotus root, CL: crunchy lotus root, CK: background).

## Data Availability

The data presented in this study are openly available in [National Center for Biotechnology Information] at [https://www.ncbi.nlm.nih.gov/, accessed on 12 June 2025], reference number [PRJNA1275841, PRJNA1275856].
